# Progressive myocardial metabolic dysfunction after stereotactic arrhythmia radioablation for ventricular tachycardia in hypertrophic cardiomyopathy: a serial multimodality imaging case report

**DOI:** 10.1093/ehjcr/ytag142

**Published:** 2026-03-03

**Authors:** Masafumi Shimojo, Mariko Kawamura, Yasuya Inden, Shinji Naganawa, Toyoaki Murohara

**Affiliations:** Department of Cardiology, Nagoya University Graduate School of Medicine, 65 Tsurumai-cho, Showa-ku, Nagoya, Aichi 466-8550, Japan; Department of Cardiovascular Research and Innovation, Nagoya University Graduate School of Medicine, 65 Tsurumai-cho, Showa-ku, Nagoya, Aichi 466-8550, Japan; Department of Radiology, Nagoya University Graduate School of Medicine, 65 Tsurumai-cho, Showa-ku, Nagoya, Aichi 466-8550, Japan; Department of Cardiology, Nagoya University Graduate School of Medicine, 65 Tsurumai-cho, Showa-ku, Nagoya, Aichi 466-8550, Japan; Department of Radiology, Nagoya University Graduate School of Medicine, 65 Tsurumai-cho, Showa-ku, Nagoya, Aichi 466-8550, Japan; Department of Cardiology, Nagoya University Graduate School of Medicine, 65 Tsurumai-cho, Showa-ku, Nagoya, Aichi 466-8550, Japan

**Keywords:** Stereotactic arrhythmia radioablation, Ventricular tachycardia, Hypertrophic cardiomyopathy, Myocardial injury, Cardiac imaging, Case report

## Abstract

**Background:**

Stereotactic arrhythmia radioablation (STAR) for ventricular tachycardia (VT) has emerged as a promising treatment for patients in whom catheter ablation is challenging or unsuccessful; however, its underlying mechanisms remain unclear, particularly with respect to early antiarrhythmic effects.

**Case summary:**

A 51-year-old man with hypertrophic cardiomyopathy exhibited extensive mid-septal scar on contrast-enhanced cardiac magnetic resonance imaging (CE-MRI) and suffered from refractory VT. The VT was presumed to originate from the mid-septal scar with preferential conduction towards the outflow region. Stereotactic arrhythmia radioablation was performed, delivering 25 Gy to the core scar and 20 Gy to the surrounding wall and outflow region. Ventricular tachycardia episodes decreased immediately after STAR and disappeared within 3 months, but recurred 24 months later. Serial multimodality imaging provided novel insight into the mechanism: progressive metabolic dysfunction (reduced iodine-123-beta-methyl-p-iodophenyl-pentadecanoic acid uptake), and wall motion deterioration began soon after irradiation. The progressive decline in R-wave amplitude in the shock lead also suggested ongoing myocardial injury. These changes occurred without corresponding alterations in perfusion (thallium-201 chloride) or fibrosis (CE-MRI). Motion-phase analysis revealed a septal contraction delay before STAR that resolved at 1 month but evolved into a heterogeneous pattern by 1 year and further deteriorated by 2 years.

**Discussion:**

Myocardial irradiation with 20–25 Gy can induce progressive, non-ischaemic myocardial injury with limited fibrotic change over a prolonged period. This injury may exert short-term antiarrhythmic effects but could potentially create new arrhythmogenic substrates in the long term. Careful consideration is required when irradiating normal myocardium.

Learning points

- The early antiarrhythmic effect of stereotactic arrhythmia radioablation for ventricular tachycardia may be mediated by non-ischaemic myocardial injury with limited fibrotic change.
- Myocardial injury can occur even at a 20 Gy dose, underscoring the need for caution when planning large treatment volumes that include healthier myocardium.

## Introduction

Stereotactic arrhythmia radioablation (STAR) for ventricular tachycardia (VT) has emerged as a promising option for patients in whom catheter ablation (CA) has failed or is not feasible; however, its therapeutic mechanisms remain poorly understood.^1–5^ Although radiation-induced tissue injury and subsequent fibrosis were initially considered the primary mechanisms,^2,3,6^ clinical observations have demonstrated antiarrhythmic effects that occur far earlier than the expected development of fibrosis.^4,7^ Recent experimental studies have proposed that early effects may involve improved myocardial conduction rather than tissue injury, a concept that contrasts with the traditional understanding.^8–10^ Here, we report a case of STAR for VT in a patient with hypertrophic cardiomyopathy (HCM) in which serial imaging suggested that myocardial injury occurred much earlier than previously assumed, providing new insights into the temporal characteristics of STAR-related tissue response.

## Summary figure

**Figure:**
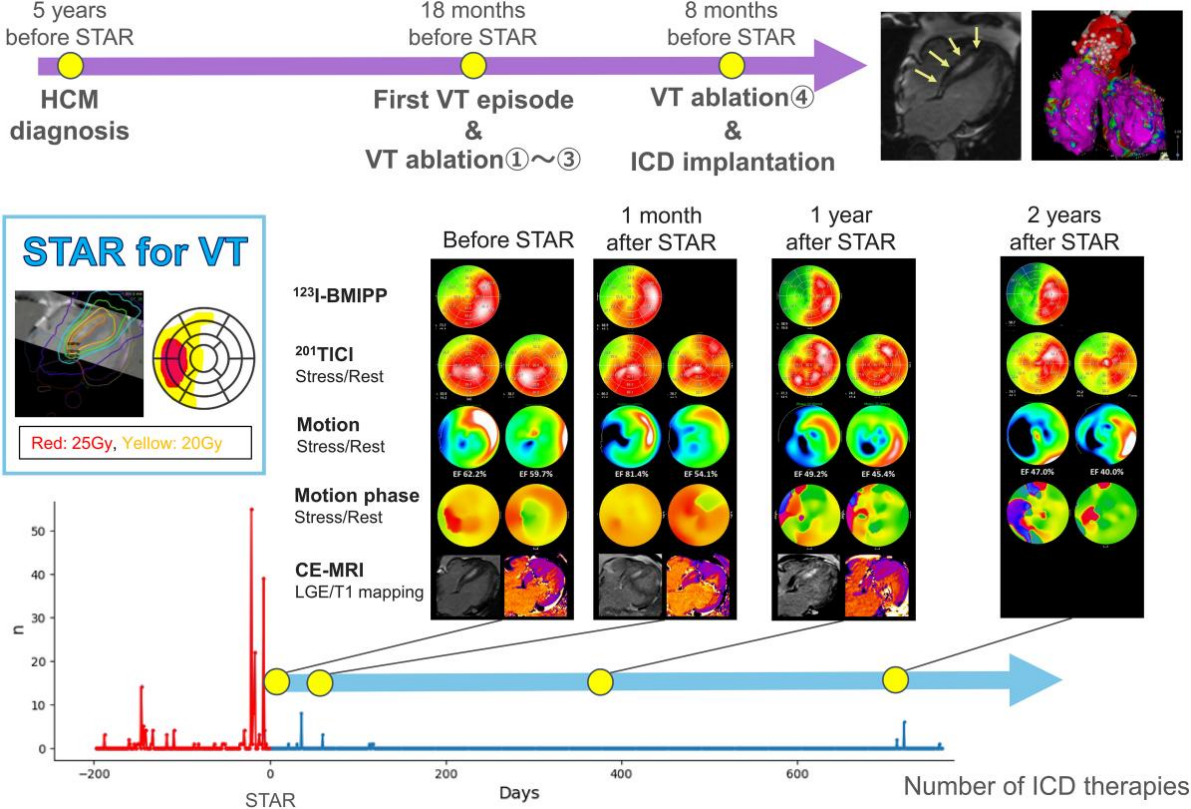


## Case presentation

**Figure 1:**
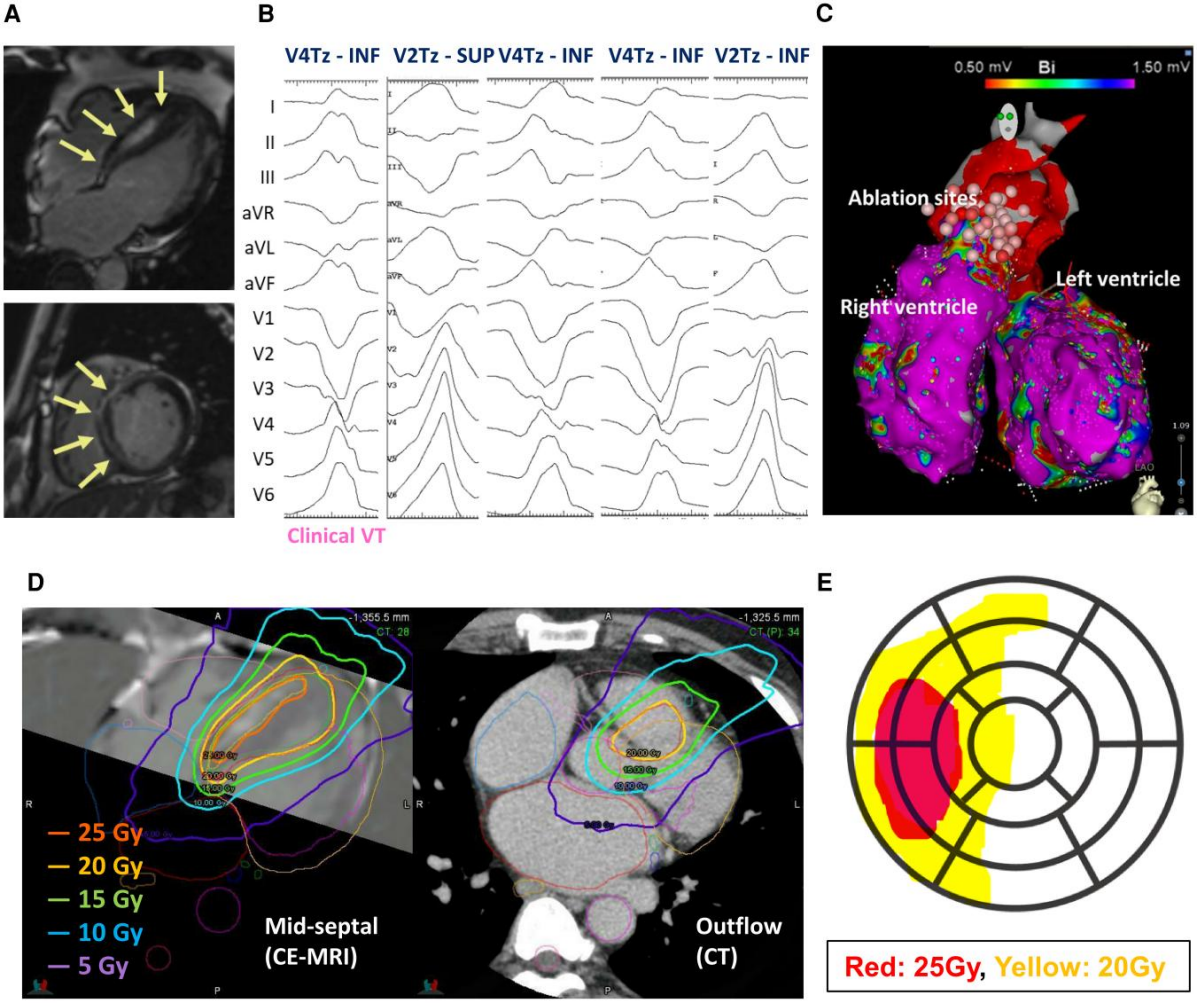
(*A*) CE-MRI. Arrows indicate areas of LGE. (*B*) Twelve-lead electrocardiogram of VTs induced during ablation. (*C*) Voltage map and ablation sites. (*D*) Radiation dose distribution (left, septal region; right, outflow region). (*E*) Schematic of the 25 and 20 Gy irradiation fields projected onto the 17-segment model. CE-MRI, contrast-enhanced cardiac magnetic resonance imaging; CT, computed tomography; INF, inferior axis; LGE, late gadolinium enhancement; SUP, superior axis; Tz, precordial transitional zone; VT, ventricular tachycardia.

A 51-year-old man with HCM, diagnosed in 2018, remained asymptomatic until April 2022, when he presented with chest pain accompanied by diaphoresis and was diagnosed with VT, for which he underwent CA. Despite three CAs targeting the left and right coronary cusps and both ventricular outflow tracts, VT recurred, and he was referred to our institution. In February 2023, CA and implantable cardioverter-defibrillator (ICD) implantation were performed. Pre-procedural contrast-enhanced cardiac magnetic resonance imaging (CE-MRI) showed mid-septal late gadolinium enhancement (LGE) of the hypertrophied septum (*Figure 1A*). The clinical VT demonstrated a V4 transition with an inferior axis, while other inducible VTs showed various precordial transitions and axes (*Figure 1B*), suggesting a mid-septal scar origin. During CA, both ventricles exhibited normal endocardial voltage, and radiofrequency energy was applied to the earliest activation site of the inducible clinical VT; however, it was ineffective (*Figure 1C*). Details of CA are shown in Supplementary material online, *Figure S1*.

Despite treatment with amiodarone 200 mg and bisoprolol 2.5 mg, the patient continued to experience frequent ICD therapies, and STAR using the CyberKnife® system (Accuray Inc., USA) was performed in October 2023. The treatment was conducted as a specified clinical study approved by the institutional review board. The VTs were thought to originate from the mid-septal arrhythmogenic scar but exit preferentially towards the outflow region. Therefore, the target was planned to cover both the mid-septal scar and the outflow region. Because including the entire transmural wall containing LGE-positive myocardium would have resulted in an excessively large volume, dose planning was as follows: 25 Gy to the LGE-positive core and 20 Gy to the remaining LGE-positive-containing wall and the outflow, without margin expansion (*Figure 1D*). The 25 Gy volume was 32.2 mL, and the combined 20/25 Gy volume was 133 mL. The 25 and 20 Gy irradiation fields projected onto the 17-segment model are shown in *Figure 1E*.

**Figure 2:**
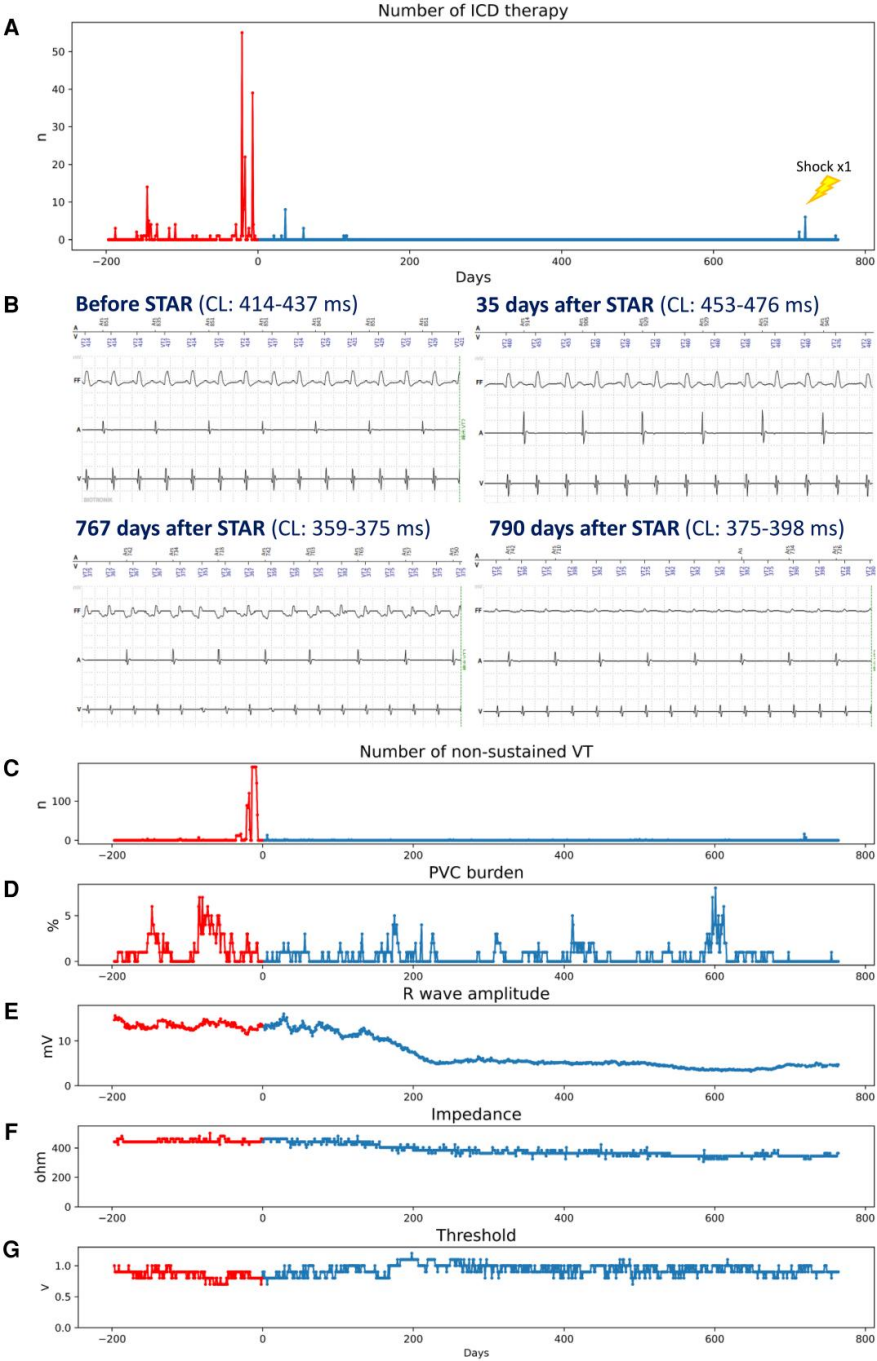
(*A*) Number of ICD therapies. Shock delivery occurred only in the marked episodes, and all therapies were anti-tachycardia pacing. (*B*) ICD recording during a VT episode. (*C*) Number of non-sustained VT episodes. (*D*) Per cent of PVCs per day. (*E*) R-wave amplitude. (*F*) Shock lead impedance. (*G*) Ventricular pacing threshold. CL, cycle length; ICD, implantable cardioverter-defibrillator; PVC, premature ventricular contraction; STAR, stereotactic arrhythmia radioablation; VT, ventricular tachycardia.

**Figure 3:**
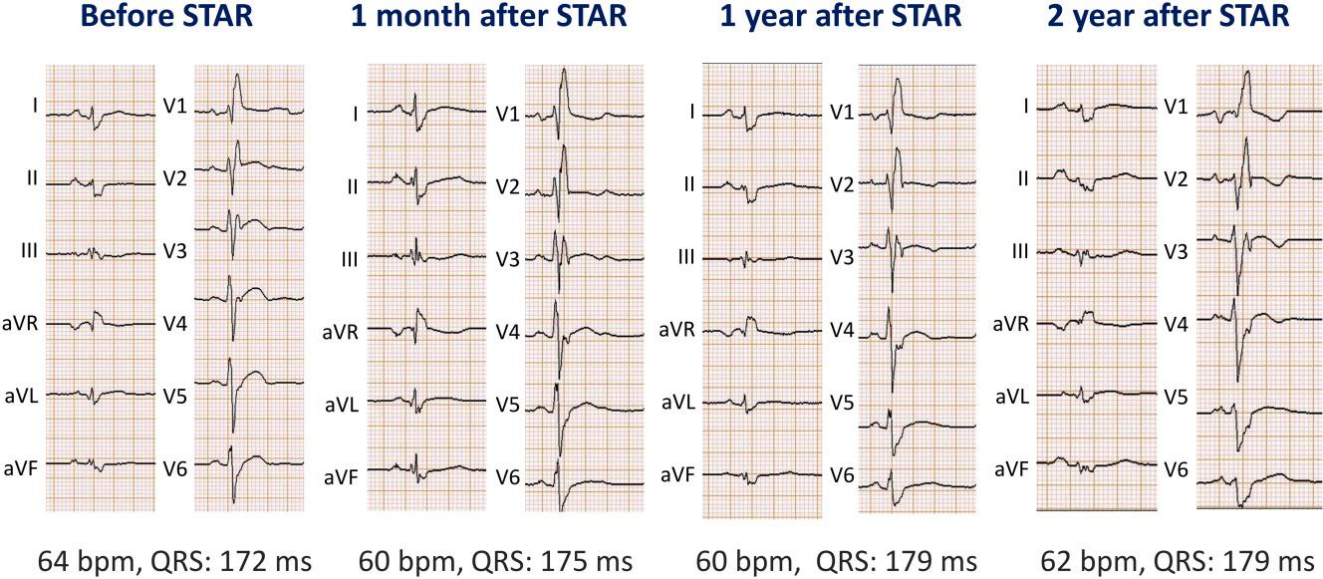
Serial 12-lead electrocardiograms. STAR, stereotactic arrhythmia radioablation.

Arrhythmic events and ICD lead parameters were collected daily through remote monitoring (Home Monitoring®, Biotronik SE & Co. KG, Germany) for 6 months before and 25 months after STAR. The ICD lead tip was fixed at the right ventricular septum. Implantable cardioverter-defibrillator therapies decreased immediately after STAR and were absent beyond 3 months; however, appropriate ICD therapies, including shock delivery, recurred 24 months later (*Figure 2A*). One month after STAR, the VT showed a slightly longer cycle length but similar morphology to pre-STAR, whereas the recurrent VT at 24 months had a different morphology and a shorter cycle length (*Figure 2B*). Non-sustained VT decreased in parallel with VT episodes (*Figure 2C*), while premature ventricular contraction burden remained almost unchanged (*Figure 2D*). The R-wave amplitude gradually declined and reached a plateau at around 7 months (*Figure 2E*). Ventricular pacing thresholds and impedance showed similar trends, with thresholds increasing and impedance decreasing until stabilizing at ∼7 months (*Figure 2F* and *G*). The surface electrocardiogram showed no remarkable changes (*Figure 3*).

**Figure 4:**
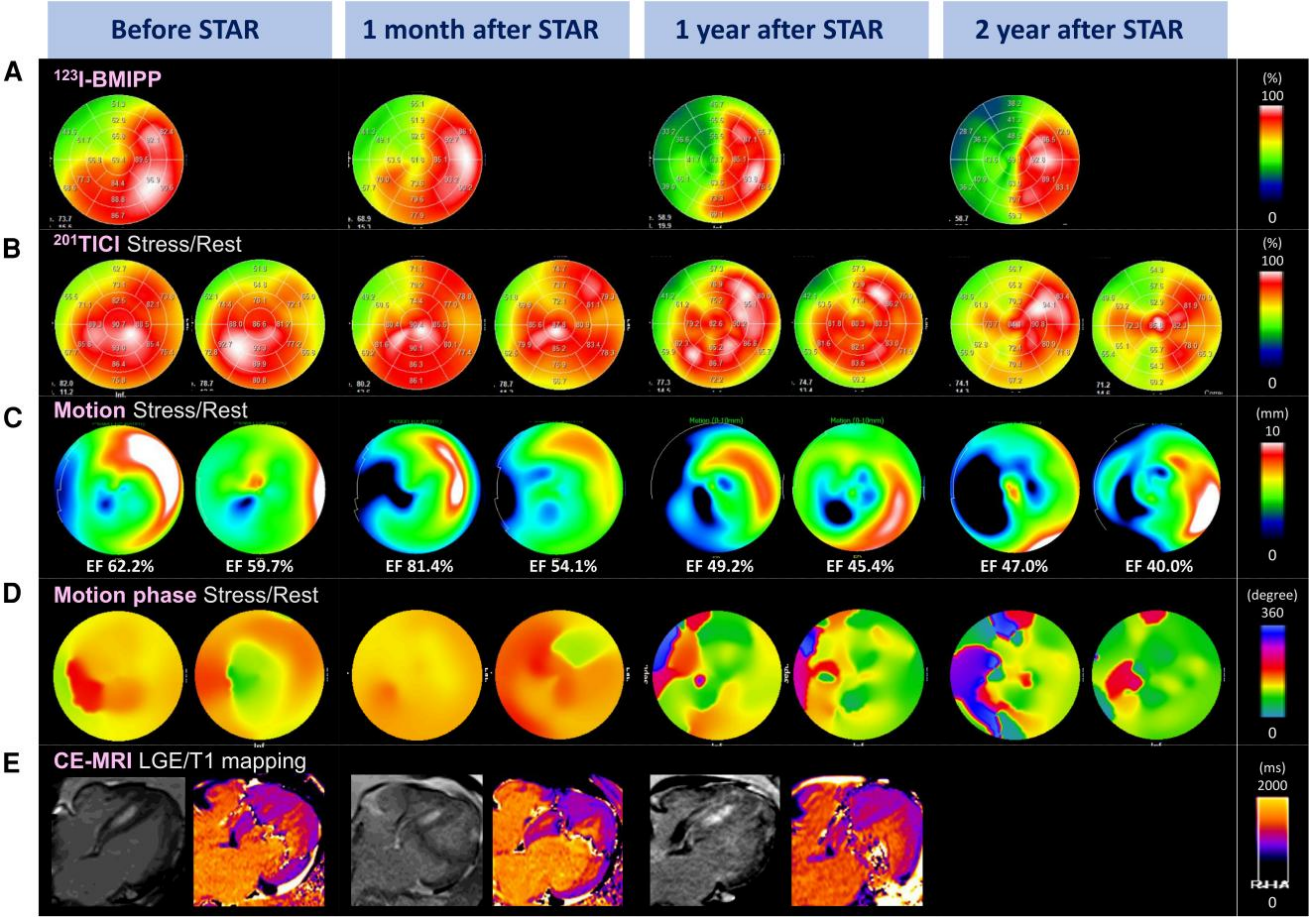
Serial imaging changes. (*A*) ^123^I-BMIPP uptake. (*B*) ^201^TlCl uptake (right, stress; left, rest). (*C*) Left ventricular wall motion (right, stress; left, rest). (*D*) Phase analysis of left ventricular motion (right, stress; left, rest). (*E*) MRI (left: LGE; right: T1 mapping). CE-MRI, contrast-enhanced cardiac magnetic resonance imaging; EF, ejection fraction; ^123^I-BMIPP, iodine-123-beta-methyl-p-iodophenyl-pentadecanoic acid; LGE, late gadolinium enhancement; STAR, stereotactic arrhythmia radioablation; ^201^TlCl, thallium-201 chloride.

Dual single-photon emission computed tomography using thallium-201 chloride (^201^TlCl) and iodine-123-beta-methyl-p-iodophenyl-pentadecanoic acid (^123^I-BMIPP), along with CE-MRI, was performed before STAR and at 1 month, 1 year, and 2 years. ^123^I-BMIPP imaging demonstrated progressive reduction in tracer uptake within the irradiated area (*Figure 4A*), whereas ^201^TlCl uptake showed no apparent change on either stress or rest imaging (*Figure 4B*). Quantitative gated single-photon emission computed tomography (QGS) revealed progressive regional motion deterioration in the irradiated region (*Figure 4C*). Motion-phase analysis using Heart Risk View software (HRV-F, Nihon Medi-Physics, Japan) showed a septal contraction delay during stress before STAR, which disappeared at 1 month but evolved into a heterogeneous contraction pattern by 1 year, and further deteriorated by 2 years (*Figure 4D*). On CE-MRI, no substantial change was observed in the areas of LGE or T1-mapping signal intensity (*Figure 4E*).

At 25 months after STAR, the patient continued regular outpatient follow-up without major complications, except for a mild pericardial effusion observed at 6 months. Detailed temporal changes in physical status, mental status, and medication are summarized in Supplementary material online, *Figure S2*.

## Discussion

In this case, STAR demonstrated an early antiarrhythmic effect (*Figure 2A*), accompanied by early-onset, long-term progressive myocardial injury in the irradiated region. Ventricular tachycardia suppression by STAR may involve not only indirect effects, such as autonomic modulation, but also direct injury to the VT isthmus and altered conduction properties. ^123^I-BMIPP uptake within the irradiated region decreased over time (*Figure 4A*). Although early post-STAR changes may reflect oedema or intramyocardial haemorrhage,^11^ they likely represent an early phase of progressive, irreversible myocardial injury rather than a transient reversible process. In contrast, ^201^TlCl uptake under both rest and stress conditions was preserved (*Figure 4B*), suggesting that the myocardial injury was non-ischaemic and reflected radiation-induced damage, possibly mediated by free radicals or apoptosis. There were no findings suggestive of marked fibrotic progression (*Figure 4E*), consistent with prior studies.^6,10,11^ Progressive wall motion deterioration within the irradiated area on QGS (*Figure 4C*) and the gradual decline in R-wave amplitude (*Figure 2E*) further supported progressive myocardial injury. Although R-wave reduction may partly reflect lead–myocardium interface effects, its temporal trend suggested that myocardial injury began immediately after STAR and stabilized around 7 months, consistent with previously reported apoptotic changes following myocardial irradiation.^12,13^

The HRV-F findings also provided important insights into the antiarrhythmic mechanism. The VT was presumed to originate from a septal scar, and pre-STAR phase analysis demonstrated delayed septal activation during stress, which may have served as the arrhythmogenic substrate. This delay disappeared 1 month after STAR, potentially explaining the early antiarrhythmic effect. However, more than 1 year after STAR, heterogeneous phase patterns emerged in the irradiated region (*Figure 4D*). Given that the recurrent VT differed from the pre-STAR morphology (*Figure 2B*), a new arrhythmogenic substrate may have developed during the chronic phase due to progressive myocardial injury.

These findings may be unique to HCM; however, similar phenomena could potentially occur in other aetiologies as well. The detection of progressive injury in this case may have been facilitated by the hypertrophied myocardium, which differs from the thinned myocardium often seen in other cardiomyopathies and provides sufficient spatial resolution for detection on both MRI and nuclear imaging. Given that a single-fraction STAR dose of 25 Gy, or even 20 Gy, can induce progressive myocardial injury, irradiation of normal myocardium should be minimized whenever possible. In particular, although transmural irradiation of the ventricular wall has been widely accepted,^14^ the target should be carefully defined when a large proportion of normal myocardium is included in the treatment region.

## Supplementary Material

ytag142_Supplementary_Data

## Data Availability

The data underlying this article will be shared upon reasonable request to the corresponding author.

## Lead author biography

Masafumi Shimojo specializes in clinical cardiac electrophysiology. He graduated from Nagoya University School of Medicine in 2011 and worked in the Department of Cardiology at Nagoya Ekisaikai Hospital and Anjo Kosei Hospital. Since 2022, he has been serving as a Designated Assistant Professor in the Department of Cardiovascular Research and Innovation at Nagoya University. His research primarily focuses on clinical electrophysiology and CA.

## Notes

**Consent:** Written informed consent for the submission and publication of this case was obtained from the patient in accordance with the COPE guidelines.
