# Supplementary material for: Progressive myocardial metabolic dysfunction after stereotactic arrhythmia radioablation for ventricular tachycardia in hypertrophic cardiomyopathy: a serial multimodality imaging case report
Source: Eur Heart J Case Rep. 2026 Mar 3;10(3):ytag142. doi: 10.1093/ehjcr/ytag142 (PMC13034031; doi:10.1093/ehjcr/ytag142)
Supplement: ytag142_Supplementary_Data [file ytag142_supplementary_data.docx]

**Supplementary Figure S1**


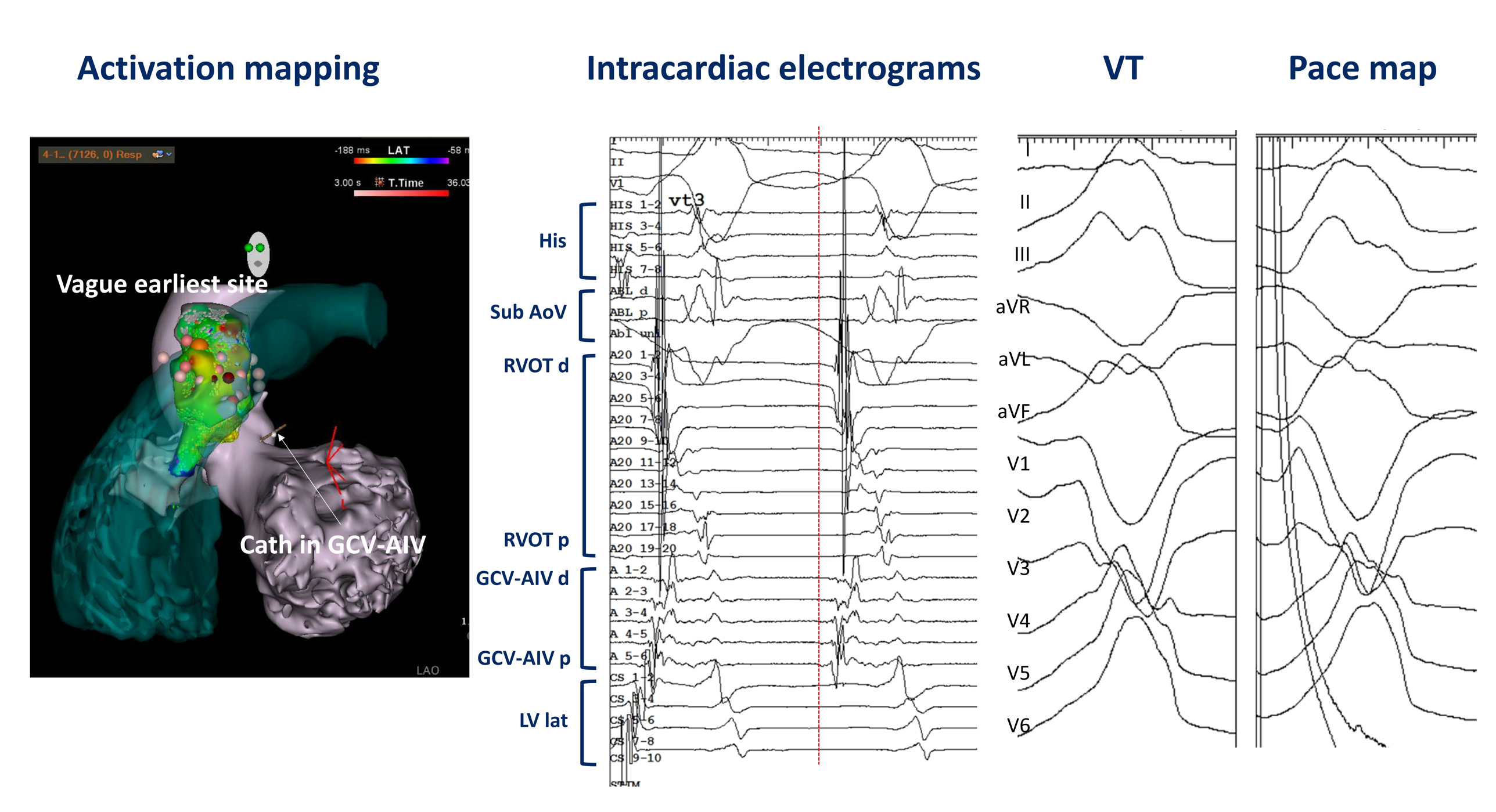


Details of the ablation procedure at our institution. Substrate mapping of both the left and right endocardium revealed no abnormal electrograms. We therefore performed activation mapping of an inducible ventricular tachycardia (VT) that was considered to represent the clinical VT. The earliest activation was identified in the right ventricular outflow tract (RVOT); however, rather than a discrete focal earliest site, a relatively broad region demonstrated vaguely early activation.

During VT, intracardiac electrogram mapping showed that although the RVOT was the earliest site among regions accessible to the ablation catheter, fractionated low-amplitude electrograms recorded by electrodes positioned in the great cardiac vein–anterior interventricular vein (GCV–AIV) were the earliest overall. Nevertheless, these potentials preceded the QRS onset by no more than approximately the onset itself. Although not shown in the figures, electrograms recorded from the aortic cusps were also evaluated, but showed minimal early activation.

The best pace map obtained from the RVOT exhibited a QRS morphology similar to that of the target VT; however, complete reproduction of the VT morphology, including the R-wave patterns in the precordial leads, was not achieved. Entrainment pacing at this site demonstrated constant fusion, with a post-pacing interval approximately 35 ms longer than the tachycardia cycle length.

Radiofrequency ablation was performed in the RVOT during VT; however, it had no effect on the VT. Taken together, these findings suggest that although a reentrant mechanism was likely, the endocardial RVOT represented only an exit site. Considering these electrophysiological findings in conjunction with the cardiac MRI results, we hypothesized that the VT circuit was located in the deep interventricular septum, an area inaccessible to conventional mapping. At the referring institution, ablation had primarily targeted the aortic cusps, which we believe reflected subtle differences in exit sites rather than the true origin.

Because there was no electrophysiological or imaging evidence to suggest an epicardial circuit, epicardial approach was not performed. In addition, because the exact VT origin could not be precisely localized, bipolar ablation was not performed.

**Supplementary Figure S2**





Clinical findings and course. Implantable cardioverter-defibrillator (ICD) therapy burden (as shown in Figure 2a) and patient activity were assessed using BIOTRONIK Home Monitoring. Patient activity increased before VT recurrence two years after stereotactic arrhythmia radioablation (STAR). Physical condition was evaluated using the New York Heart Association (NYHA) functional class and the SF-36 physical component score, both of which showed no substantial changes before and after STAR or during the chronic phase. In contrast, mental status assessed by the SF-36 mental component score improved following STAR, coinciding with effective suppression of ventricular tachycardia (VT). This improvement in mental status was considered to have contributed to the subsequent increase in patient activity.

Serial echocardiography demonstrated a gradual decline in left ventricular ejection fraction (LVEF), measured using the Simpson method, accompanied by a parallel increase in plasma B-type natriuretic peptide (BNP) levels. The types of medications and their daily dosages are summarized in the bottom panel. At the referring hospital, mexiletine, verapamil, and bisoprolol had been prescribed, but their therapeutic effects were considered insufficient. After catheter ablation at our institution, mexiletine and verapamil were discontinued, and amiodarone was initiated. At the 6-month follow-up after STAR, an increase in BNP levels and pericardial effusion (possibly related to STAR) were observed, leading to the addition of furosemide and spironolactone, followed by candesartan for heart failure management. The VT recurrence two years after STAR was considered to have been triggered by excessive physical workload resulting from a dissociation between impaired cardiac function and improved mental status. At that time, lifestyle modification was reinforced, and empagliflozin was added to intensify heart failure therapy. No further VT recurrence has been observed since then.
